# The Epidemic in Spain

**Published:** 1918-11

**Authors:** 


**The Epidemic in Spain.** *British Medical Journal*, June 8, 1918. Editorial.

Widespread epidemic of an acute catarrhal infection in Spain is reported to have caused 700 deaths in 10 days; but if the number of cases has been as large as reported, the case mortality must have been very low.

The disease attacks the respiratory rather than the abdominal organs; relapses frequently occur within a few days; and, although the disease is clearly of the character of influenza, bacteriological examination has not resulted in the discovery of the influenza bacillus, but has revealed an organism described as the parameningococcus.

Although an epidemic of meningococcus carriers may reach a very high percentage among contacts, we are not cognizant of any previous outbreak of cerebro-spinal fever in any degree comparable in extent to the epidemic in Spain. It is obvious that further bacteriological information must be awaited.

